# Optimized segmented regression models for the transition period of intervention effects

**DOI:** 10.1186/s41256-023-00312-3

**Published:** 2023-07-24

**Authors:** Xiangliang Zhang, Kunpeng Wu, Yan Pan, Rong Yin, Yi Zhang, Di Kong, Qi Wang, Wen Chen

**Affiliations:** 1 Department of Medical Statistics, School of Public Health, Sun Yat-sen University, Guangzhou, China; 2 Center for Migrant Health Policy, Sun Yat-sen University, Guangzhou, China

**Keywords:** Segmented regression, Transition period, Intervention evaluation, Cumulative distribution functions, Distribution patterns

## Abstract

**Background:**

The interrupted time series (ITS) design is a widely used approach to examine the effects of interventions. However, the classic segmented regression (CSR) method, the most popular statistical technique for analyzing ITS data, may not be adequate when there is a transitional period between the pre- and post-intervention phases.

**Methods:**

To address this issue and better capture the distribution patterns of intervention effects during the transition period, we propose using different cumulative distribution functions in the CSR model and developing corresponding optimized segmented regression (OSR) models. This study illustrates the application of OSR models to estimate the long-term impact of a national free delivery service policy intervention in Ethiopia.

**Results:**

Regardless of the choice of transition length ($L$) and distribution patterns of intervention effects, the OSR models outperformed the CSR model in terms of mean square error (MSE), indicating the existence of a transition period and the validity of our model’s assumptions. However, the estimates of long-term impacts using OSR models are sensitive to the selection of *L*, highlighting the importance of reasonable parameter specification. We propose a data-driven approach to select the transition period length to address this issue.

**Conclusions:**

Overall, our OSR models provide a powerful tool for modeling intervention effects during the transition period, with a superior model fit and more accurate estimates of long-term impacts. Our study highlights the importance of appropriate statistical methods for analyzing ITS data and provides a useful framework for future research.

**Supplementary Information:**

The online version contains supplementary material available at 10.1186/s41256-023-00312-3.

## Background

The interrupted time series (ITS) is most commonly used to evaluate the effects of interventions such as quality improvement programs or health policies [1, 2] and is a powerful quasi-experimental design [2] especially when randomized controlled trials are impossible, unethical, or not feasible [3–5].

The most popular statistical methodology for ITS time-series data of interventions is the classic segmented regression (CSR) [6, 7], which is a potent method for accounting for underlying trends and has a high ability to infer causation [8]. To distinguish between the pre- and post-intervention phases, the CSR model restricts the interruption to a predetermined time point in the outcome time series [9]. The impact of interventions is frequently portrayed in CSR as instant, immediate, and leapfrogging at a fixed point [7].

However, the immediate effect of the intervention may not always hold, which is inconsistent with CSR assumptions. Many studies have shown that there may be a transition period between the pre- and post-intervention phases [2, 10, 11]. Because interventions may be effective over a prolonged period, or there may be a brief period of adjustment before interventions’ lasting impacts on the outcome time series become apparent [2]. First, interventions may have been introduced over time. For example, in England's 2012 Health and Social Care Act, the Clinical Commissioning Groups first took on the task in April 2012 and eventually took over full budget responsibility in March 2013; therefore, there was a one-year transition period before the intervention was fully implemented [12]. Second, interventions may require a brief adjustment period (training required for intervention implementation). For instance, the Clinical Nurse Leader intervention program launched by the American Association of Colleges of Nursing, aimed to enhance healthcare quality, and the nurses involved required training for several months before the program was formally implemented [13]. The nurses put what they learned into practice in their clinical work during the training period, which allows the intervention to generate an adjustment period before its fully functional [9]. Similar intervention training was scheduled for the implementation of the free maternal health services policy in Kenya [14]. Such training is necessary for interventions to improve healthcare quality and equity.

In the aforementioned examples, the effects of interventions may be released during the transition period. That is, the intervention gradually shows the effect after the predetermined interruption (within the usually defined “post-intervention phase”). However, the CSR model fails to model this period precisely. For the time points of the transition period, the CSR model either ignores them to model the entire outcome time series directly or removes them and then models the remaining time points [6, 15]. For example, in evaluating the effect of pay for performance on hypertension in the United Kingdom, the period corresponding to the stepwise implementation of the intervention was excluded from the interrupted time-series analysis [16]. As researchers chose to exclude the transition period, this censoring (removing the transition period) not only leaves out data but could also distort parameter estimations on the effect of interventions [17]. If an intervention is found to be effective based on an inaccurate or biased estimation of its effects, resources may be allocated to scale up the intervention, which could potentially waste resources, and divert attention from other effective interventions [18, 19].

To solve this problem, we propose an optimized segmented regression (OSR) model to capture different distribution patterns of the intervention effects during the transition period using probability density functions (PDFs) types. We then utilize the corresponding cumulative distribution functions (CDFs) of the above PDFs to model the effects of the interventions during the transition period and introduce them into the CSR model. The transition period commenced when the intervention was initially introduced, and the length of the transition period reflects the time horizon over which interventions are effective or the length of the required training time for intervention implementation. Furthermore, CDFs can manifest in various forms, reflecting different distributions of intervention effects. In this study, we discuss four common distributions, namely uniform distribution, normal distribution, log-normal distribution (right-skewed distribution), and log-normal flip distribution (left-skewed distribution), to characterize the possible distributions of intervention effects during the transition period.

In this study, we first describe the steps of the optimized model. Then taking the evaluation of the free delivery service policy in five Ethiopian health centers as an empirical study example [20], we estimated the long-term impact of the free delivery service policy using the CSR model and OSR models with different CDFs. In this process, we suggest a possible data-driven approach for selecting the length of the transition period using the mean squared error (MSE) as a measure of the goodness of fit of the OSR model. By comparing the estimated long-term impacts of the models, we illustrated the advantages and disadvantages of the optimized models and their applicability.

## Models

### Classic segmented regression (CSR)

1 $$Y_{t} = \beta_{0} + \beta_{1} \times time + \beta_{2} \times intervention + \beta_{3} \times {post}\text{-}{time} + \varepsilon_{t} .$$

${Y}_{t}$ is the value of the outcome series at time point $t$. $time$ is an indicator variable of the time point ($time = 1,2,3, \ldots ,T_{e}$) and spans the first and last observation points. ${T}_{0}$ is the time point at which the intervention is implemented (nominal intervention time), and ${T}_{e}$ is the length of the entire time series. A dummy variable, $intervention$, was used to represent the implementation of the intervention. The dummy variables 0 and 1 values represent pre- and post-intervention, respectively. The time elapsed after the nominal implementation of the intervention is monitored using the ${post}\text{-}{time}$ indicator variable. The value of ${post}\text{-}{time}$ is first set to 1 during the post-implementation phase and then increases over time (${post}\text{-}{time} = 1, 2, 3, \ldots , \;T_{e} - T_{0} < T_{e}$). The random error term for time point $t$ is${\varepsilon }_{t}$. Before the implementation, the outcome series' baseline trend is depicted by ${\beta }_{1}$. ${\beta }_{2}$ reflects the instant effect of the intervention on ${Y}_{t}$. The long-term impact of the intervention consists in the change in the trend of the outcome time series (slopes), represented by${\beta }_{3}$. The matrix expression of Eq. (1) is:

2 $${\varvec{Y}}={{\varvec{X}}}_{{\varvec{C}}{\varvec{S}}{\varvec{R}}}\boldsymbol{*}{\varvec{\beta}}+{\varvec{\varepsilon}};\boldsymbol{ }{\varvec{\beta}}={\left[{\beta }_{0},{\beta }_{1},{\beta }_{2},{\beta }_{3}\right]}^{T},$$

where

$${{\varvec{X}}}_{{\varvec{C}}{\varvec{S}}{\varvec{R}}}=\left[\begin{array}{cccc}1& 1& 0& 0\\ \vdots & \vdots & \vdots & \vdots \\ 1& {T}_{0}& 0& 0\\ 1& {T}_{0}+1& 1& 1\\ \vdots & \vdots & \vdots & \vdots \\ 1& {T}_{e}& 1& {T}_{e}-{T}_{0}\end{array}\right].$$

### Optimized segmented regression (OSR)

In the optimized model (Eq. 3), we model the transition period using different forms of CDFs as follows:

3 $$Y_{t} =\, \beta_{0} + \beta_{1} \times time + \beta_{2} \times F\left( t \right) \times intervention + \beta_{3} \times F\left( t \right) \times {post}\text{-}{time} + \varepsilon_{t} .$$

The piecewise function $F\left(t\right)$ is:

4 $$F\left( t \right) = \left\{ {\begin{array}{*{20}l} {CDF\left( {t - T_{0} } \right),} \hfill & {T_{0} < t \le T_{2} ;} \hfill \\ {1,} \hfill & {T_{2} < t} \hfill \\ \end{array} } \right..$$

where ${T}_{0}$ is the nominal intervention time and has the same definition as the CSR model. ${T}_{2}$ is the end time of the transition period, ${T}_{2}={T}_{0}+L$, where $L$ stands for “transition length”. The effect of the intervention is assumed to last from ${T}_{0}$ (first implementation) to ${T}_{2}$ (fully valid): the transition period $\left[{T}_{0},{T}_{2}\right]$. $CDF\left(t\right)$ represent the CDFs of the different distribution patterns of the intervention effect during the transition period.

The variable assignments (${time}$, $intervention$, and ${post}\text{-}{time}$) of the optimized model and the meanings of the corresponding coefficients were the same as the CSR model. The matrix expression of Eq. (3) is:

5 $${\varvec{Y}}={{\varvec{X}}}_{{\varvec{O}}{\varvec{S}}{\varvec{R}}}\boldsymbol{*}{\varvec{\beta}}+{\varvec{\varepsilon}};\boldsymbol{ }{\varvec{\beta}}={\left[{\beta }_{0},{\beta }_{1},{\beta }_{2},{\beta }_{3}\right]}^{T},$$

where

$${{\varvec{X}}}_{{\varvec{O}}{\varvec{S}}{\varvec{R}}}=\left[\begin{array}{cccc}1& 1& 0& 0\\ \vdots & \vdots & \vdots & \vdots \\ 1& {T}_{0}& 0& 0\\ 1& {T}_{0}+1& 1*CDF\left(1\right)& 1*CDF\left(1\right)\\ \vdots & \vdots & \vdots & \vdots \\ 1& {T}_{0}+L& 1*CDF\left(L\right)& L*CDF\left(L\right)\\ 1& {T}_{0}+L+1& 1& L+1\\ \vdots & \vdots & \vdots & \vdots \\ 1& {T}_{e}& 1& {T}_{e}-{T}_{0}\end{array}\right].$$

#### Distribution patterns of intervention effects—CDFs

**Fig. 1:**
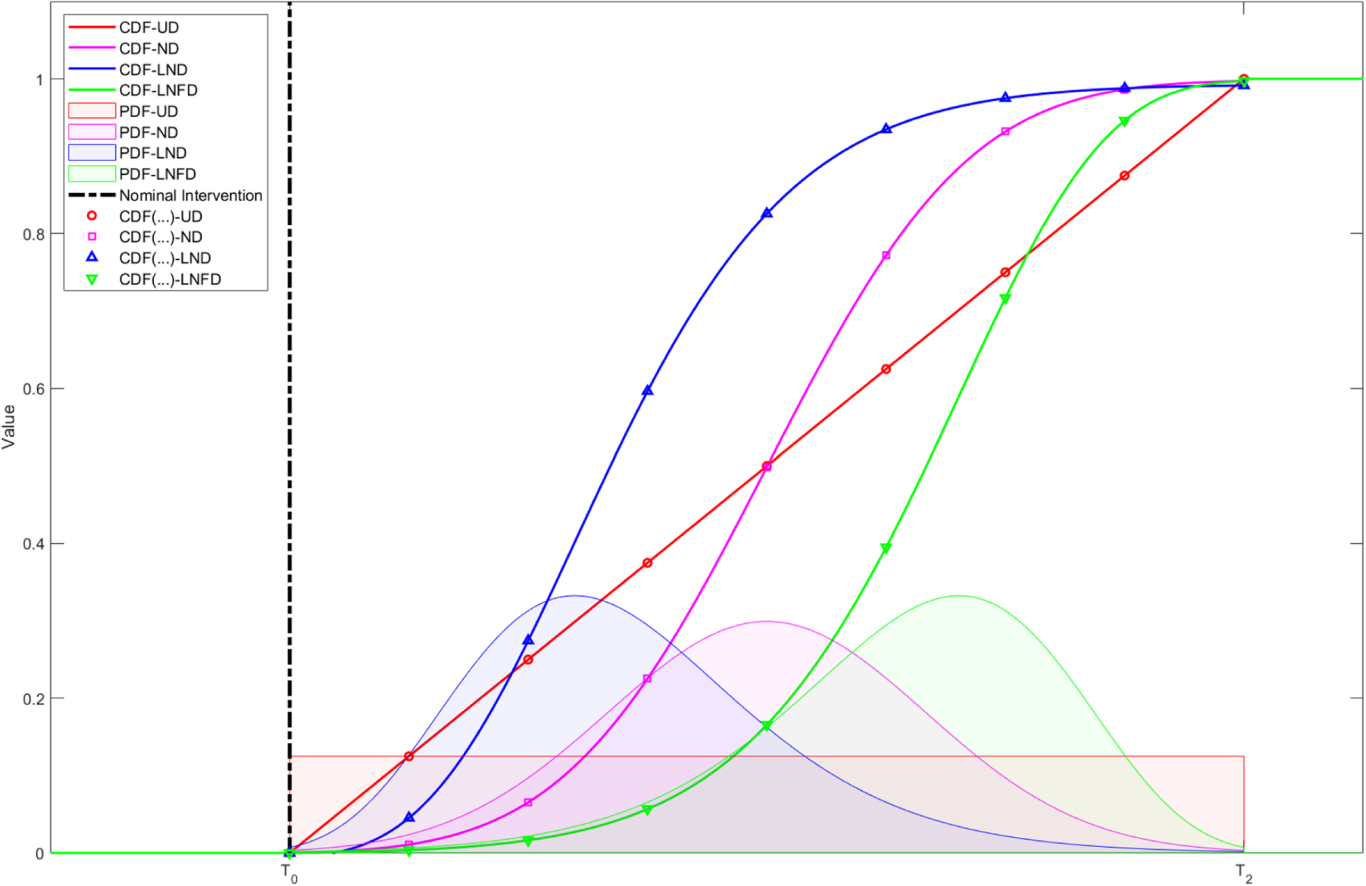
Schematic diagram of CDFs and corresponding PDFs for different distribution patterns of intervention effects

$CDF\left(t\right)$ are the CDFs of the corresponding PDFs for the different distribution patterns of the intervention effect during the transition period. The PDFs represent how the effect of the intervention is distributed during the transition period $[{T}_{0},{T}_{2}]$ and the values of the corresponding CDFs taken at specific points are used for modeling, that is, $CDF\left(1\right),\dots ,CDF\left(L\right)$. In this study, we mainly discuss the common distributions: (1) uniform distribution, (2) normal distribution, (3) log-normal distribution (right-skewed distribution), and (4) log-normal flip distribution (left-skewed distribution). The CDFs and the corresponding PDFs are shown in Fig. 1.

**Fig. 2:**
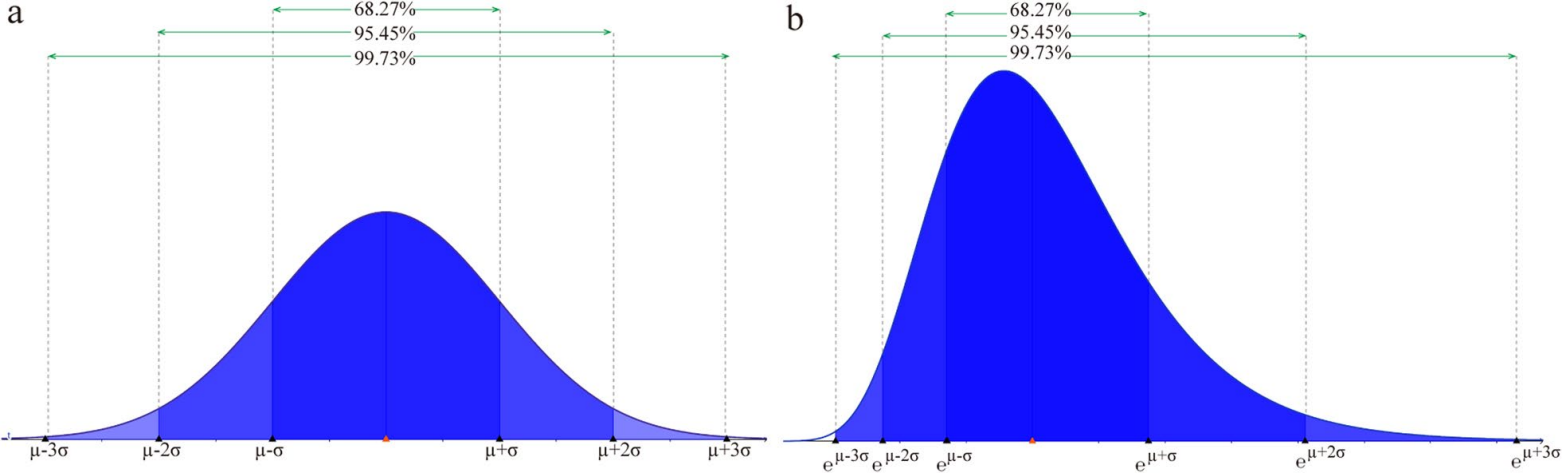
Schematic diagram of the truncated probability distribution for the normal and lognormal distributions

For the normal and log-normal distributions, their PDFs are respectively defined in the domain $[- \infty , + \infty ]$ and $[0, + \infty ]$. We truncated the PDFs so that we can describe the effect of the intervention at a fixed interval $[{T}_{0},{T}_{2}]$. The probability of occurrence of a fixed interval can be determined by integrating the PDF. For the normal and log-normal distributions, we chose $(\mu -3\sigma ,\mu +3\sigma$) and $({e}^{\mu -3\sigma },{e}^{\mu +3\sigma })$, respectively, to truncate them such that the probability of occurrence in the fixed interval is up to 99.97%. Matching the truncated interval to our assumed time range $[{T}_{0},{T}_{2}]$, we have $$\left\{\begin{array}{c}{T}_{0}=\mu -3\sigma \\ {T}_{2}=\mu +3\sigma \end{array}\right.$$ for the normal distribution and $$\left\{\begin{array}{c}{T}_{0}={e}^{\mu -3\sigma }\\ {T}_{2}={e}^{\mu +3\sigma }\end{array}\right.$$ for the log-normal distribution. The intervention was essentially fully effective at $[{T}_{0},{T}_{2}]$. The truncated intervals of the normal and log-normal distributions are shown in Fig. 2. For the log-normal flip distribution, we only needed to apply an axisymmetric flip transformation to the truncated log-normal distribution. The log-normal and log-normal flip distributions represented the right-skewed and the left-skewed distributions, respectively, and accordingly indicated that intervention effects are concentrated in the front or the back part of the transition period $[{T}_{0},{T}_{2}]$.

##### Uniform distribution pattern (UD)

For a uniform distribution in the interval $[{T}_{0},{T}_{2}]$, its PDF and CDF are:

$$\left\{ {\begin{array}{*{20}l} {PDF_{UD} \left( t \right) = \frac{1}{L};} \hfill \\ {CDF_{UD} \left( t \right) = \frac{t}{L};} \hfill \\ \end{array} } \right. \quad 1 \le t \le L.$$

Then $${{\varvec{C}}{\varvec{D}}{\varvec{F}}}_{UD}=\left[{CDF}_{UD}\left(1\right),\dots ,{CDF}_{UD}\left(L\right)\right]=\left[\frac{1}{L},\dots ,1\right]$$*.*

##### Normal distribution pattern (ND)

For a normal distribution, its PDF and CDF are:

$$\left\{ {\begin{array}{*{20}l} {PDF_{ND} \left( x \right) = \frac{1}{{\sqrt {2\pi } \sigma }}\exp \left( { - \frac{{(x - \mu )^{2} }}{{2\sigma^{2} }}} \right);} \hfill \\ {CDF_{ND} \left( x \right) = \mathop \int \nolimits_{ - \infty }^{x} PDF_{ND} \left( y \right)dy;} \hfill \\ \end{array} } \right.\quad - \infty \le x \le + \infty .$$

The PDF of the normal distribution is an infinite integral; we truncated its PDF and calculated its mean $\mu$ and standard deviation $\sigma$, as $$\left\{\begin{array}{l}\sigma =\frac{L}{6}; \\ \mu ={T}_{0}+3\sigma .\end{array}\right.$$ At one specific time point $t$,

$$CDF_{ND} \left( t \right) = \mathop \int \nolimits_{ T_{0 } }^{T_{0 }+t} PDF_{ND} \left( x \right)dx;\quad 1 \le t \le L.$$

Then $${\varvec{CDF}}_{ND}=\left[{CDF}_{ND}\left(1\right),\dots ,{CDF}_{ND}\left(L\right)\right]=\left[{\int}_{{T}_{0}}^{{T}_{0}+1}{PDF}_{ND}\left(x\right)dx,\dots ,{\int }_{{T}_{0}}^{{T}_{2}}{PDF}_{ND}\left(x\right)dx\right]$$*.*

##### Log-normal distribution pattern (LND)

For a log-normal distribution, in its definition domain $[0,+\infty ]$, its PDF and CDF are:

$$\left\{ {\begin{array}{*{20}l} {PDF_{LND} \left( x \right) = \frac{1}{{x\sigma \sqrt {2\pi } }}exp\left( { - \frac{{\left( {\operatorname { ln }x - \mu } \right)^2 }}{{2\sigma^{2} }}} \right);} \hfill \\ {CDF_{LND} \left( x \right) = \int\nolimits_{0}^{x} {PDF_{LND}(y) dy = \int\nolimits_{0}^{x} {\frac{1}{{y\sigma \sqrt {2\pi } }}e^{ - \frac { ( \operatorname { ln } y - \mu ) ^ { 2 } } { 2 \sigma ^ { 2 } } } dy = \int\nolimits_{0}^{x} {\frac{1}{\sqrt \pi }e^{ - \frac { ( \operatorname { ln } y - \mu ) ^ { 2 } } { 2 \sigma ^ { 2 } } } d\left[ {\frac{{\left( {\operatorname { ln }y - \mu } \right)}}{\sqrt{2} \sigma }} \right]} } } } \hfill \\ \end{array} .} \right.$$

When an upper limit exists, this integral cannot be solved using algebraic operations; its integral is usually expressed in the form of an error function as follows.

$$CDF_{LND} \left( x \right) = \frac{1}{2}\left\{ {1 + erf\left[ {\frac{{\left( {\ln x - \mu } \right)}}{\sqrt 2 \sigma }} \right]} \right\};erf\left( x \right) = \frac{2}{\sqrt \pi }\mathop \smallint \limits_{0}^{x} e^{{ - y^{2} }} dy.$$

Assuming that ${CDF}_{LND}\left(x\right)=\frac{1}{2}\left\{1+erf\left[\frac{(\ln x-\mu )}{\sqrt{2}\sigma }\right]\right\}$ = 0.5, with the integral symmetry, the median coordinate of the log-normal distribution is $x={e}^{\mu }$*.* The corresponding coordinate interval where the sample falls near the median with a distance of $3\sigma$ standard deviation is $({e}^{\mu -3\sigma },{e}^{\mu +3\sigma })$*.* Here, we used the same strategy as that for the truncated PDFs in the normal distribution. However, the log-normal distribution is skewed; thus, we additionally set its skewness ratio, which is defined by the ratio of the release time of the half effect of the intervention in a total transition period of intervention, i.e., $Ratio=\frac{{e}^{\mu }-{e}^{\mu -3\sigma }}{{e}^{\mu +3\sigma } - {e}^{\mu -3\sigma }}$. For instance, in the context of a 12-session training course spanning three months, the parameter $Ratio$=$\frac{1}{3}$ of the lognormal distribution implies that half of the training sessions were concluded within the initial month, specifically six sessions. The degree of skewness, denoted by the $Ratio$, depends on the skewness of the actual intervention effect during the transition period $[{T}_{0},{T}_{2}]$. Correspondingly, we truncated its PDF and calculated its mean $\mu$ and standard deviation $\sigma$, as $$\left\{\begin{array}{l}{e}^{\mu }-{e}^{\mu -3\sigma }=Ratio*L;\\ {e}^{\mu +3\sigma }-{e}^{\mu -3\sigma }=L.\end{array}\right.$$ At one specific time point $t$,

$${CDF}_{LND}\left(t\right)=\frac{1}{2}\left\{1+\mathit{erf}\left[\frac{\left(\ln ({T}_{0}+t)-\mu \right)}{\sqrt{2}\sigma }\right]\right\}, 1\le t\le L.$$

Then $$\begin{aligned}{{\varvec{CDF}}}_{LND}&=\left[{CDF}_{LND}\left(1\right),\dots ,{CDF}_{LND}\left(L\right)\right]\\ &=\left[\frac{1}{2}\left\{1+\mathit{erf}\left[\frac{\left(\ln({T}_{0}+1)-\mu \right)}{\sqrt{2}\sigma }\right]\right\},\dots ,\frac{1}{2}\left\{1+\mathit{erf}\left[\frac{\left(\ln{T}_{2}-\mu \right)}{\sqrt{2}\sigma }\right]\right\}\right] \end{aligned}$$.

##### Log-normal flip distribution pattern (LNFD)

**Fig. 3:**
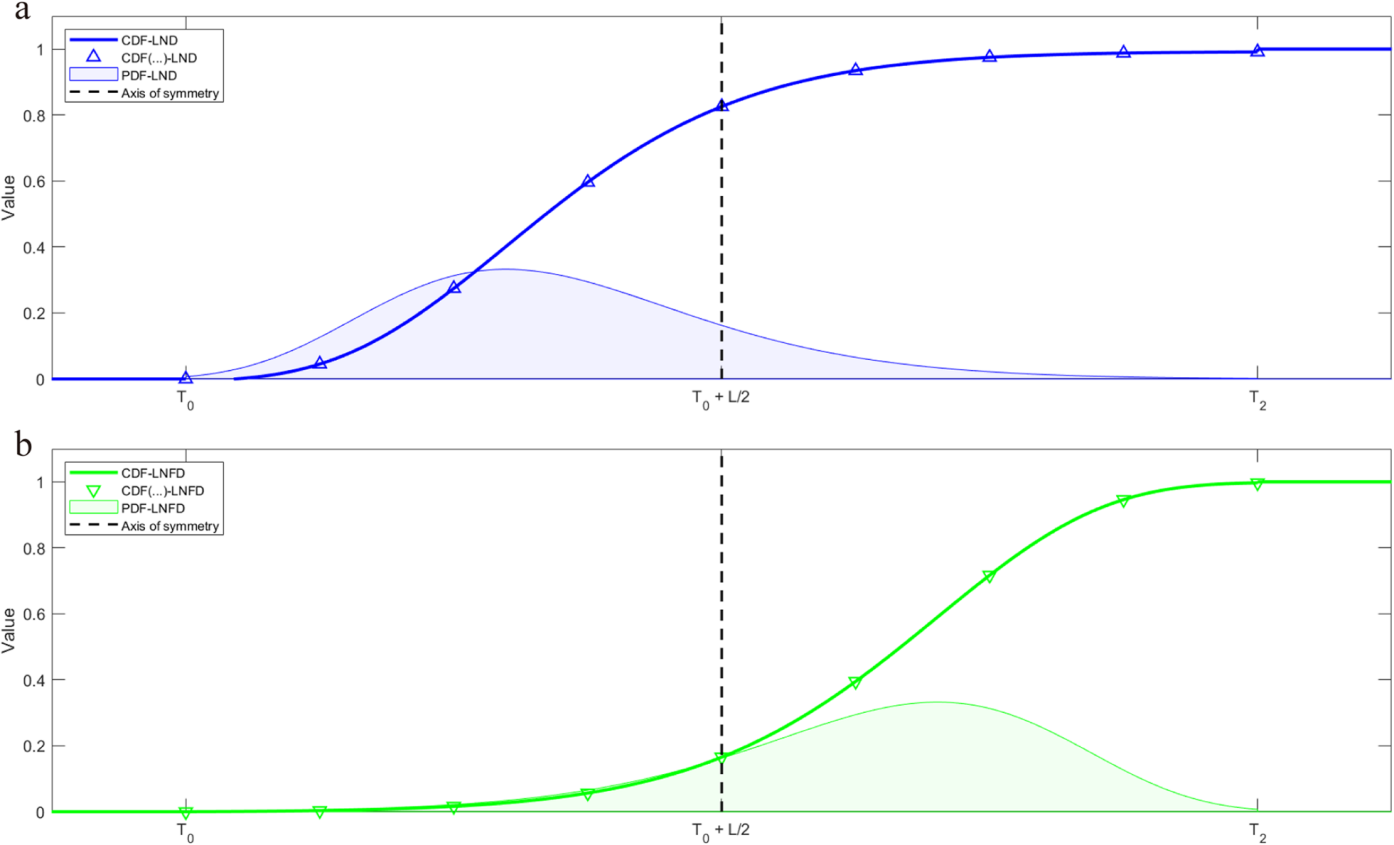
PDFs and CDFs of log-normal distribution and log-normal flip distribution

For the log-normal flip distribution, we applied only an axisymmetric flip transformation to the truncated log-normal distribution. We chose the midpoint coordinates $x={T}_{0}+\frac{L}{2}$ of the transition period as the axis of symmetry to perform the axisymmetric flip transformation of the log-normal distribution, allowing us to obtain the log-normal flip PDF and integrate it to obtain its CDF. The schematic diagram of the axisymmetric flip transformation is shown in Fig. 3.

According to the symmetry of the axisymmetric flip transformation, then we have

$$\begin{aligned} {\varvec{CDF}}_{LNDF} & = \left[ {CDF_{LNDF} \left( 1 \right), \ldots ,CDF_{LNDF} \left( {L - 1} \right),CDF_{LNDF} \left( L \right)} \right] \\ & = \left[ {CDF_{LND} \left( L \right) - CDF_{LND} \left( {L - 1} \right), \ldots ,CDF_{LND} \left( L \right) - CDF_{LND} \left( 1 \right),CDF_{LND} \left( L \right)} \right]. \\ \end{aligned}$$

By modeling the four above-mentioned distribution patterns of the intervention effect, we developed four OSR branching models: OSR-UD, OSR-ND, OSR-LND, and OSR-LNFD.

#### Length of the transition period

In most cases, the length $L$ of the transition period and the distribution pattern of the intervention effect are determined by the implementation process. When there was no information about the implementation process, we used a data-driven approach to select $L$ for the above four distribution patterns of intervention effect and described the application process of the optimized model.

First, we set the maximum possible range for $L$ selection, that is, the ${L}_{m}$ (${L}_{m}=\max L$). We then applied the optimized OSR model directly to all scenarios ($L=0, 1, 2,\dots ,{L}_{m}$), and ${L}_{m}+1$ scenarios for each OSR branching model for a total of $4\times ({L}_{m}+1)$ scenarios. $L=0$ corresponds to the CSR model; that is, there is no transition period. For the different distribution patterns of the intervention effect, we selected the value of $L$ corresponding to the minimum MSE in all scenarios.

## Application data analysis

### Data description

In this study, we used raw data from a published research article [20] titled *‘Effect of Implementing a Free Delivery Service Policy on Women’s Utilization of Facility-Based Delivery in Central Ethiopia: An Interrupted Time Series Analysis’*, to test and compare the CSR model and our optimized models. The raw data are provided in the supplementary file of the above research article and can be downloaded directly from the *Journal of Pregnancy* [20].

**Fig. 4:**
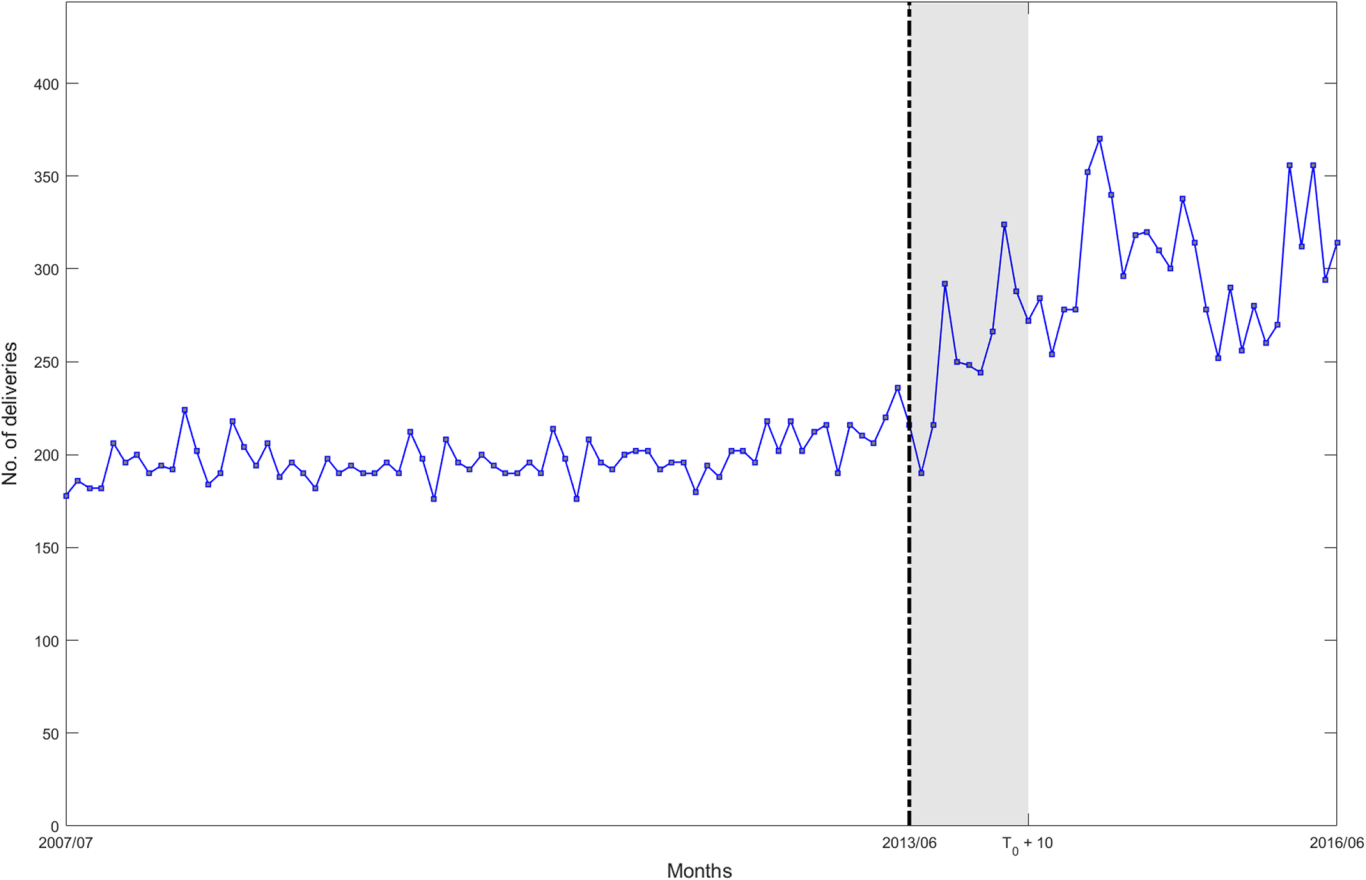
Outcome time series of total monthly births. July 2007 and June 2016 are the start and end time points, respectively, of the time series, while June 2013 represents the nominal intervention time point (free delivery services policy). The shaded area in the figure represents an example scenario of the transition period $[{T}_{0},{T}_{2}]$ corresponding to $L=10$

In Ethiopia, facility delivery services were not widely available or used. To encourage mothers to give birth in health facilities, the Ethiopian government implemented a policy of free delivery services in all public health facilities in July 2013. The government established a primary health care facility in the East Shewa administrative region where the national free delivery service intervention was implemented in all public health centers. Primary-level care has been established by the government, which consists of health posts, health centers (HCs), and primary/district hospitals. Five HCs (Adama, Awashmelkasa, Bishoftu, Modjo, and Walinchity) with complete data from the previous nine years were chosen. For the nine years from July 2007 to June 2016, 108 data points were available, including facility-based usage of delivery services (72 pre- and 36 post-intervention phases). The total number of monthly births in the five HCs mentioned above served as the outcome variable (Fig. 4).

The Ethiopian government implemented a national free delivery service. After the formal intervention implementation (2013/07), the Ethiopian government undertook a series of works to get this policy intervention fully off the ground, such as purchasing emergency vehicles, increasing the number of beds in health facilities, and related delivery equipment for women, and training relevant health care workers. Additionally, most pregnant women do not enjoy the benefits immediately if the gap between policy advocacy and public awareness is considered. Meanwhile, even if women in the Shewa region learned about the free delivery service policy when the intervention was formally implemented (2013/07) and became pregnant immediately, they only gave birth after nearly ten months. Therefore, the intervention effect cannot be fully interrupted at the time point when the intervention is implemented, as assumed by the CSR model. Thus, this intervention is considered an ideal application case for the optimized models.

### Selection of the length of the transition period

**Fig. 5:**
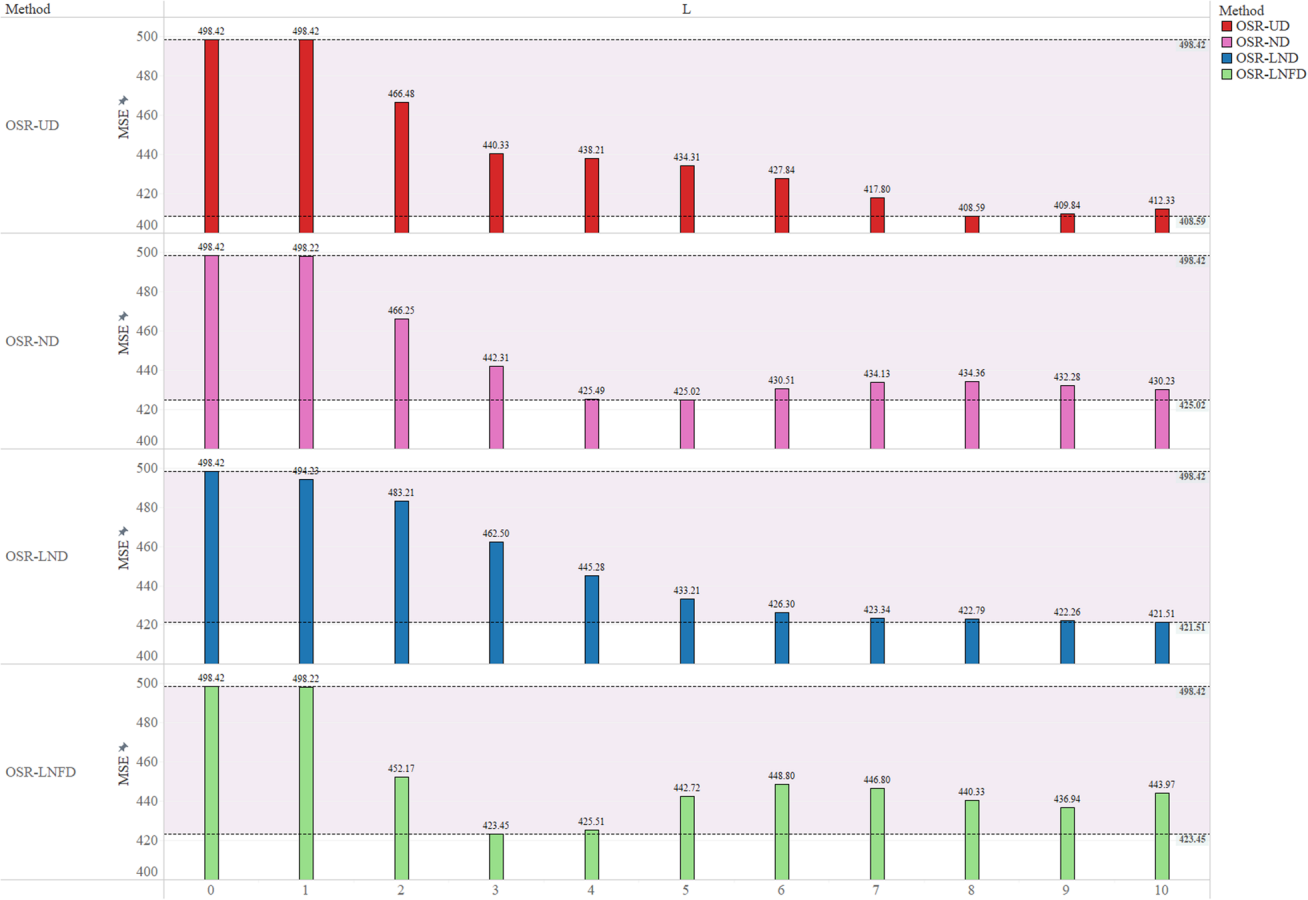
Mean squared errors under different distribution patterns of the intervention effect. The heights of the bars indicate the magnitude of the mean-squared error (MSE). The horizontal axis represents the different lengths of the transition period $L$. Different colors represent different methods. The shaded parts of the figure represent the maximum and minimum ranges of the different methods for the corresponding MSE. When $L=0$, the OSR model degenerates into the CSR model

Considering the gap between policy advocacy and public awareness and the length of a woman's pregnancy, we assumed that the maximum range of $L$ was 10 months, that is, ${L}_{m}=10$. Among the possible scenario decision sets ($L=0, 1, 2,\dots ,10$), the $L$ was selected based on the minimum MSE of the model application. For the LND pattern, we additionally assumed the $Ratio=\frac{1}{3}$, namely, the half-effect release time of the national free delivery service intervention accounted for $\frac{1}{3}$ of the total transition period. Accordingly, for the LNFD pattern, the half-effect release time of the national free delivery service intervention accounted for $\frac{2}{3}=1-\frac{1}{3}$ of the total transition period. The MSEs of the optimized model for all scenarios (all possible $L$) of the four intervention effect patterns are shown in Fig. 5.

From Fig. 5, we learned that the MSEs of the OSR models under different distribution patterns of the intervention effect were smaller than those of the CSR model ($L=0$), regardless of $L$, indicating that the OSR models fit the data better. In the different OSR models, with the increase in $L$, the change trajectories of the MSEs are different.

Taking the minimum MSE as the selection metric, different OSR models selected different transition lengths. The results of the selected $L$ for the four distribution patterns of the intervention effect and the corresponding model statistics are presented in Table 1. As shown in Fig. 5 and Table 1, the selected $L$ for the UD pattern of intervention effects was 8, that is, ${T}_{2}$ were 8 months after ${T}_{0}$. The selected $L$ for the ND, LND and LNFD patterns were 5, 10, and 3, respectively. Among the four distribution patterns of the intervention effect, the OSR-UD achieved the smallest MSE (408.5852).

**Table 1:** $L$ selected results for four distribution patterns of intervention effect

Method	*L*	MSE	*R*^2^	*F*	*P*	EEV
CSR	0	498.4159	0.8015	140.0032	< 0.001	517.5858
OSR-UD	8	408.5852	0.8373	178.4058	< 0.001	424.3000
OSR-ND	5	425.0226	0.8308	170.1654	< 0.001	441.3696
OSR-LND	10	421.5121	0.8322	171.8713	< 0.001	437.7241
OSR-LNFD	3	423.4491	0.8314	170.9266	< 0.001	439.7356

*UD*: uniform distribution; *ND*: normal distribution; *LND*: log-normal distribution; *LNFD*: log-normal flip distribution. *MSE*: mean square error. ${R}^{2}$, $F$, $P$, and EEV are the model statistics. ${R}^{2}$ is the coefficient of determination and indicates the model's goodness of fit. The $F$ is the test statistic of the F-test in the regression model, and $P$ is the corresponding p-value. EEV: estimate of error variance

In Additional file 1: Fig. S1, there were parameter estimation result planes and corresponding external studentized residuals for different distribution patterns of intervention effect, which indicated suitable fits.

In addition to MSE, the mean absolute error (MAE), mean absolute percentage error (MAPE), and median absolute deviation (MAD) can also be used as model fit metrics. The results of the $L$ selection corresponding to the minimum of the other model fit metrics are shown in the Additional file 2: Table S1 and Additional file 3: Fig. S2, Additional file 4: Fig. S3, Additional file 5: Fig. S4. Different model fit metrics may lead to different selection results for $L$.

### Results of modeling

#### Results of models with selected ${\varvec{L}}$

For intervention evaluation, the long-term impact ${\beta }_{3}$ is the most important evaluation indicator [21]. The results of the parameter estimation for the classic and four optimized models are listed in Table 2. We find heterogeneity in the parameter estimation results between the CSR and OSR models. The long-term impact estimate ${\widehat{\beta }}_{3}$ 1.4251 (95%CI: 0.6574, 2.1928) of the CSR model was higher than the estimates of the OSR model; specifically, the estimates were 0.1755 (− 0.6432, 0.9942), 0.7318 (− 0.0394, 1.5030), 0.4045 (− 0.4036, 1.2125) and 0.8751 (0.1241, 1.6260) for OSR-UD, OSR-ND, OSR-LND, and OSR-LNFD models, respectively. Compared with the OSR models, the CSR model overestimated the long-term impact ${\widehat{\beta }}_{3}$. It is worth noting that OSR-UD, OSR-ND, and OSR-LND had long-term impacts estimates greater than zero, indicating positive long-term impacts of the interventions; however, these were not statistically significant.

**Table 2:** Coefficients and 95% confidence intervals for different models

Method	*L*	${\widehat{\beta }}_{0}$ (95% CI)	${\widehat{\beta }}_{1}$ (95% CI)	${\widehat{\beta }}_{2}$ (95% CI)	${\widehat{\beta }}_{3}$ (95% CI)
CSR	0	189.3106 (178.5652,200.0561)	0.2434 (− 0.0124,0.4992)	52.8540 (34.2369,71.4712)	1.4251 (0.6574,2.1928)
OSR-UD	8	189.7346 (180.1447,199.3244)	0.2264 (0.0042,0.4486)	85.8777 (64.4468,107.3086)	0.1755 (− 0.6432,0.9942)
OSR-ND	5	189.8157 (179.9989,199.6325)	0.2228 (− 0.0061,0.4517)	71.5910 (52.4684,90.7136)	0.7318 (− 0.0394,1.5030)
OSR-LND	10	186.0201 (176.2252,195.8151)	0.2408 (0.0154,0.4662)	78.7880 (58.2046,99.3713)	0.4045 (− 0.4036,1.2125)
OSR-LNFD	3	190.1878 (180.3650,200.0105)	0.2080 (− 0.0221,0.4382)	69.0397 (50.6087,87.4706)	0.8751 (0.1241,1.6260)

*UD*: uniform distribution; *ND*: normal distribution; *LND*: log-normal distribution; *LNFD*: log-normal flip distribution

#### ${\widehat{{\varvec{\beta}}}}_{3}$ estimates for all possible scenarios of ${\varvec{L}}$

**Fig. 6:**
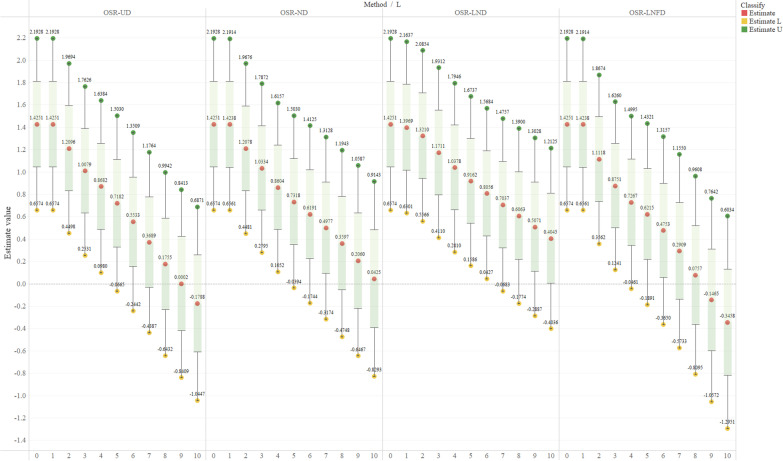
${\widehat{\beta }}_{3}$ and corresponding 95% CIs for all scenarios. The median of the box-and-whisker diagram is the estimated value ${\widehat{\beta }}_{3}$, and the lower and upper whiskers are the lower and upper limits of the 95% confidence interval of ${\widehat{\beta }}_{3}$, respectively. In the legend of the above figure, *Estimate* represents ${\widehat{\beta }}_{3}$ estimate result, and *Estimate L* and *Estimate U* represent the lower and upper limits of ${\widehat{\beta }}_{3}$ 95% CIs, respectively

We estimated the long-term impact ${\widehat{\beta }}_{3}$ of intervention effects for all possible length scenarios with OSR-UD, OSR-ND, OSR-LND, and OSR-LNFD models; the corresponding results are shown in Fig. 6. The estimates of ${\widehat{\beta }}_{3}$ were sensitive to the length of the transition period $L$. With an increase in $L$, the estimates of long-term impact ${\widehat{\beta }}_{3}$ kept decreasing for all four types of OSR models. There were slight differences between the estimates of different OSR models with the same $L$. For the outcome time series analyzed in this study, OSR-LND tended to provide the largest long-term impact estimates, whereas OSR-LNFD did the opposite. When $L$ was too large, some OSR models (OSR-UD and OSR-LNFD) estimated a negative long-term impact of the national free delivery service intervention, which was not convincing.

We presented the other coefficient estimate results of different OSR models under all possible length scenarios in the Additional file 6: Table S2, Additional file 7: Table S3, and Additional file 8: Table S4 correspond to ${\widehat{\beta }}_{0}$, ${\widehat{\beta }}_{1}$ and ${\widehat{\beta }}_{2}$, respectively. Additional file 6: Table S2 and Additional file 7: Table S3 showed that the estimates of ${\widehat{\beta }}_{0}$ and ${\widehat{\beta }}_{1}$ were almost the same for different choices of $L$. While the estimates of ${\widehat{\beta }}_{2}$ were sensitive to the length $L$ of the transition period, as shown in Additional file 8: Table S4, the estimated ${\widehat{\beta }}_{2}$ values of the different OSR models increased as $L$ increased.

## Discussion

In this study, to characterize different distribution patterns of intervention effects during the transition period, we introduced different CDFs to the CSR model and proposed the corresponding OSR-UD, OSR-ND, OSR-LND, and OSR-LNFD models. Using the national free delivery service policy intervention in Ethiopia as an empirical study, the OSR models fit the outcome time series (the total number of births per month in the five Ethiopian health centers) better than the CSR model based on the model fit metric MSE. In this process, we suggest a possible data-driven approach to select the length of the transition period for OSR models by using MSE as a fitness metric.

The existence of a transition period between the pre- and post-intervention phases is common, especially for policy interventions to improve healthcare quality and equity [22, 23]. Regardless of the $L$ choice, the MSE of the OSR models under different distribution patterns of the intervention effect was smaller than that of the CSR model, indicating that the assumption of the OSR model for the existence of the transition period was reasonable and the corresponding model optimization was more consistent with the actual characteristics of the real data.

Although OSR models fitted the data better than did the CSR model, there was heterogeneity in the long-term impact estimates ${\widehat{\beta }}_{3}$ of interventions when $L$ and the distribution pattern of the intervention effects during the transition period (UD, ND, LND, and LNFD) varied. The modeling results were especially sensitive to change in $L$. For example, in the current study, when the length of the transition period was too large, and some specific distribution patterns (UD and LNFD) were chosen, the OSR models might estimate non-convincing results based on the empirical study. In addition, some estimates of long-term impacts may not be statistically significant. Therefore, the selection of $L$ and distribution patterns of intervention effects are critical for the OSR models, which is also a difficulty in conducting the OSR analysis highlighted in this study. Notably, there could be two possible approaches for selecting $L$ and the distribution patterns: the implementation-driven and the data-driven approach.

Under the implementation-driven approach, the length of the transition period and the distribution patterns of intervention effects can be determined by the researcher according to the implementation process. The intervention implementer mastered the exact information about the intervention process [24–26]. For example, in the Ethiopian free delivery service policy study, the transition period was defined as the duration between the beginning and end of the training of medical staff; however, this was not reported or considered in the original study [20]. For the four possible distribution patterns we assumed, the most appropriate one based on the training process, such as the frequency of training and number of people trained per session, could also be defined. From this sense, the parameters set by the implementation-driven approach are in line with the intervention process and have practical significance [27, 28].

Although it is better to select $L$ and the distribution pattern of intervention effect in line with the intervention process, this information is not always accessible to the public. In this study, a data-driven approach was adopted. The choice of $L$ should strike a balance between being data-driven with minimal metrics (MSE or other possible metrics) and being simple enough to interpret from the interventional perspective of epidemiology, medicine, and policy [29]. From a purely data-driven perspective, the results of the choice of $L$ are not entirely consistent when different model-fitting metrics of goodness (e.g., MSE, MAE, MAPE, MAD) are used. Among the different metrics, confusion remains regarding their superiority. None of the indicators is inherently superior, and their relative superiority is conditional [30, 31]. The MAE is superior for Laplace errors, whereas the other metrics are preferable when the errors follow other distributions [32]. So far, the residual distribution type after applying the OSR models is unknown. Given this, we did not have a consensus on an “optimal’’ metric for selected $L$ [33]. In addition, the excessive pursuit of a minimum of one metric is most likely to result in overfitting of the model [34]. Therefore, the data-driven approach for $L$ is only one of the reference methods, and it is more appropriate to choose it according to the actual situation of the intervention.

Moreover, the uniqueness of the selected model parameters is not always necessary. Referring to the idea of the distributed lag model (DLM) [35, 36], we can describe the entire variance situation ($L$) of long-term impact estimates by specifying a series of transition lengths $L$, as illustrated in Fig. 6. Specifying a range of $L$ is one part of the sensitivity analysis [37], which is vital for judging the robustness of the corresponding estimates. In conclusion, for the selection of model parameters, our recommendation is, first, to conform to their practical meaning according to the implementation process; second, to take a possible data-driven approach; finally to calculate the estimated values of the model parameters for all possible cases and describe the entire variance situation of the estimated values.

In this study, we propose OSR models with four distribution patterns of intervention effects during the transition period and utilized them to estimate the specific values of the long-term impact of policy intervention. We examined real data and explained the modeling procedures in detail to provide practical insights into the impact of Ethiopia’s free delivery service policy intervention on the total number of births per month at the five health centers, providing new insights into the field of intervention evaluation.

A key strength and new aspect of this study is the inclusion of the intervention effects of the transition period into the model, rather than ignoring or cursorily removing the time points within the transition period. To correspond to the different distribution patterns of intervention effects during the transition period in practice, we abstracted four different forms of CDFs (UD, ND, LND, and LNFD), and proposed correspondingly different OSR models. In addition, for the selection of the length of the transition period, we suggested a data-driven selection approach, although it has some shortcomings.

Taking a specific policy intervention as an example, we conducted an empirical study to estimate the long-term impact of policy intervention. Simultaneously, we estimated and compared coefficients that reflected the long-term impacts of the intervention in the OSR model under 44 scenarios, considering the distribution patterns and $L$. This study provides a comprehensive description of the estimated values of OSR models under different parameter settings and provides a reference for analyzing their sensitivity and subsequent research.

There are a few limitations of this study. First, we did not fundamentally offer a selected method for $L$, although we suggested a possible data-driven approach. The choice of $L$ still depends more on its practical significance without its fixed paradigm. Second, we examine our OSR model using a single policy intervention dataset with different parameter settings to check the sensitivity of the estimating results. In addition, more re-examinations of the OSR models using practical datasets are encouraged to be done. Thirdly, the current OSR models are only applicable for continuous outcome variables and have difficulty adapting to categorical data. We need to further improve the OSR model to accommodate such data types.

## Conclusions

The CSR model may not always be sufficient when there is a transition period between the pre- and post-intervention phases. In such cases, our OSR models can be a potential alternative, because they use different CDFs to characterize the distribution patterns of the intervention effects during the transition period. To illustrate the effectiveness of the OSR models, we conducted an empirical study on national free delivery service policy intervention in Ethiopia. We estimated the long-term impact of policy intervention using both the CSR model and different OSR models and compared their estimated results. Our findings suggest that the OSR models, which are optimized to match the actual characteristics of the data, are powerful tools for accurately estimating the long-term impacts of interventions. These models demonstrate smaller MSEs and provide more accurate estimates of the long-term impacts than the CSR model. Our study emphasizes the importance of using appropriate statistical methods when evaluating intervention effects in the presence of a transition period.

## Supplementary Information

**Additional file 1**. **Table S1**: *L* selected results with different model fit metrics.

**Additional file 2**. **Fig. S1**: Parameter estimation result planes and corresponding external studentized residuals.

**Additional file 3.** **Fig. S2**: MAEs under different distribution patterns of intervention effect.

**Additional file 4**. **Fig. S3**: MAPEs under different distribution patterns of intervention effect.

**Additional file 5**. **Fig. S4**: MADs under different distribution patterns of intervention effect.

**Additional file 6**. **Table S2**: ${\widehat{\beta }}_{0}$ estimation results and corresponding 95% CIs.

**Additional file 7**. **Table S3**: ${\widehat{\beta }}_{1}$ estimation results and corresponding 95% CIs.

**Additional file 8**. **Table S4**: ${\widehat{\beta }}_{2}$ estimation results and corresponding 95% CIs.

## Data Availability

The data set used in the current study is completely public, and anyone can obtain it without any additional enquiry.

## Abbreviations

ITS: Interrupted time series
CSR: Classic segmented regression
OSR: Optimized segmented regression
CDF: Cumulative distribution functions
PDF: Probability density functions
UD: Uniform distribution
ND: Normal distribution
LND: Log-normal distribution
LNFD: Log-normal flip distribution
MSE: Mean square error
MAE: Mean absolute error
MAPE: Mean absolute percentage error
MAD: Median absolute deviation
DLM: Distributed lag model
