# Supplementary figures and images for: Optimized segmented regression models for the transition period of intervention effects
Source: Glob Health Res Policy. 2023 Jul 24;8:29. doi: 10.1186/s41256-023-00312-3 (PMC10364415; doi:10.1186/s41256-023-00312-3)

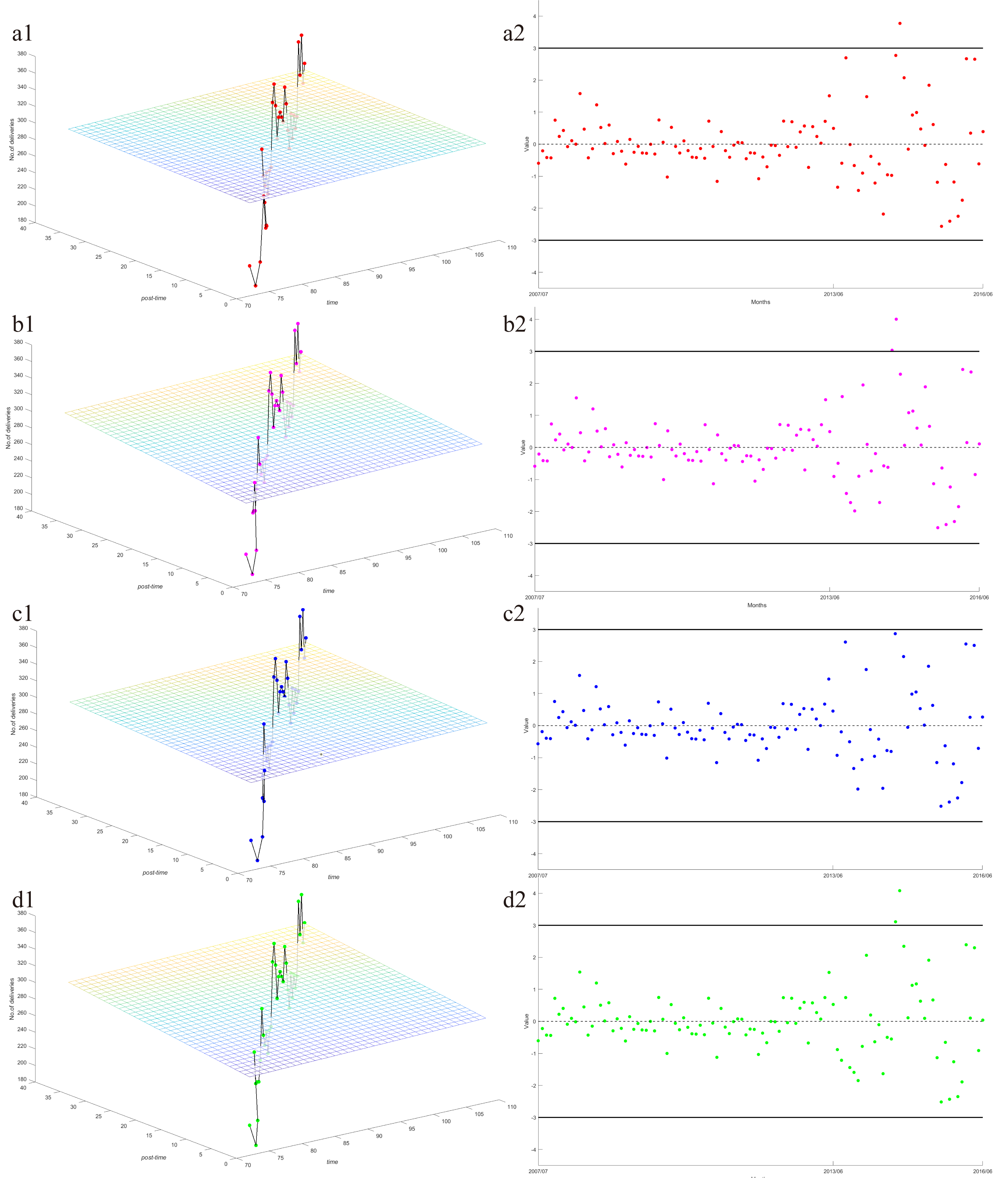

Supplement: Supplementary file 1 — Additional file 1. Table S1: L selected results with different model fit metrics. [file 41256_2023_312_MOESM1_ESM.docx]

# Additional file


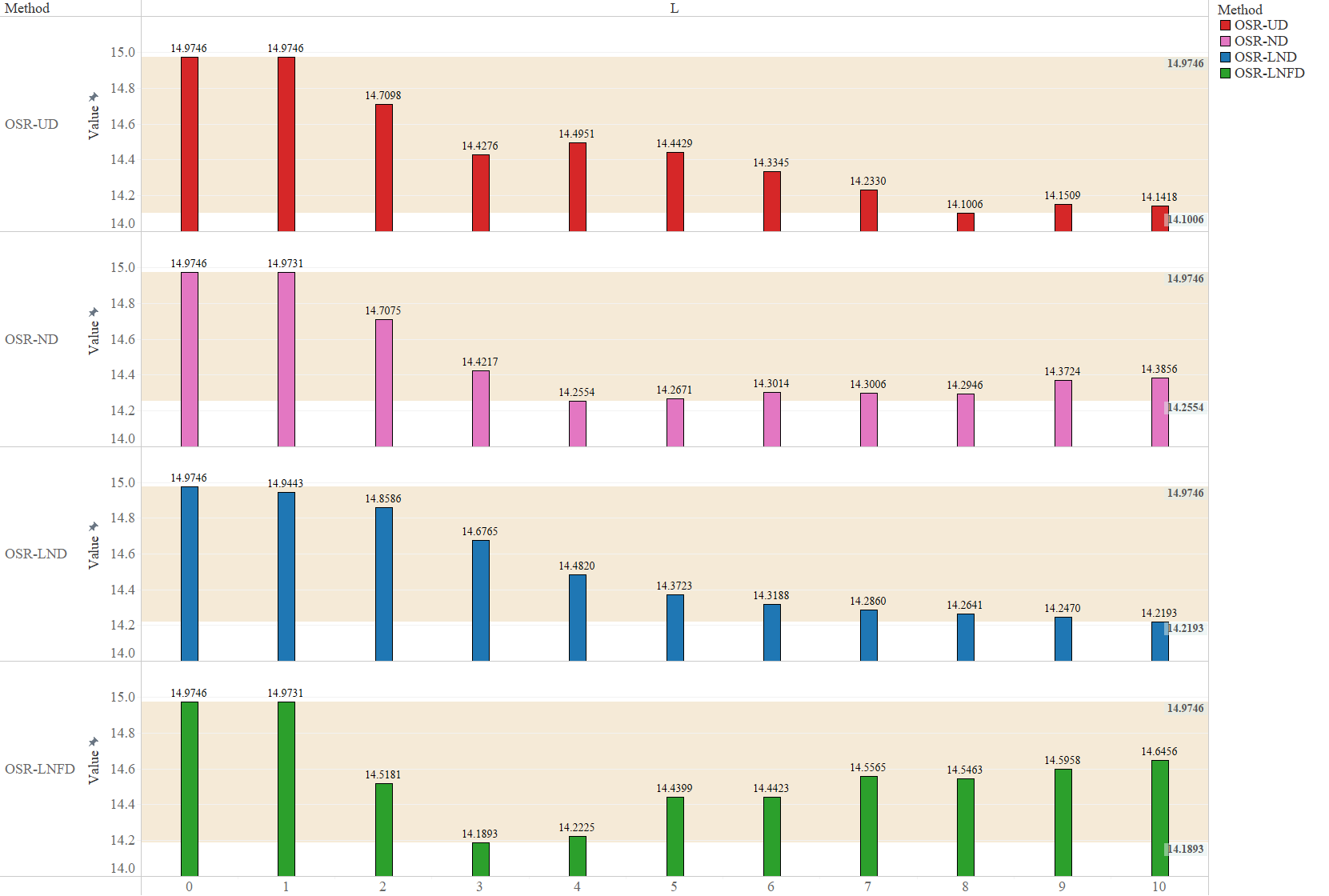

Supplement: Supplementary file 3 — Additional file 3. Fig. S2: MAEs under different distribution patterns of intervention effect. [file 41256_2023_312_MOESM3_ESM.docx]

# Additional file


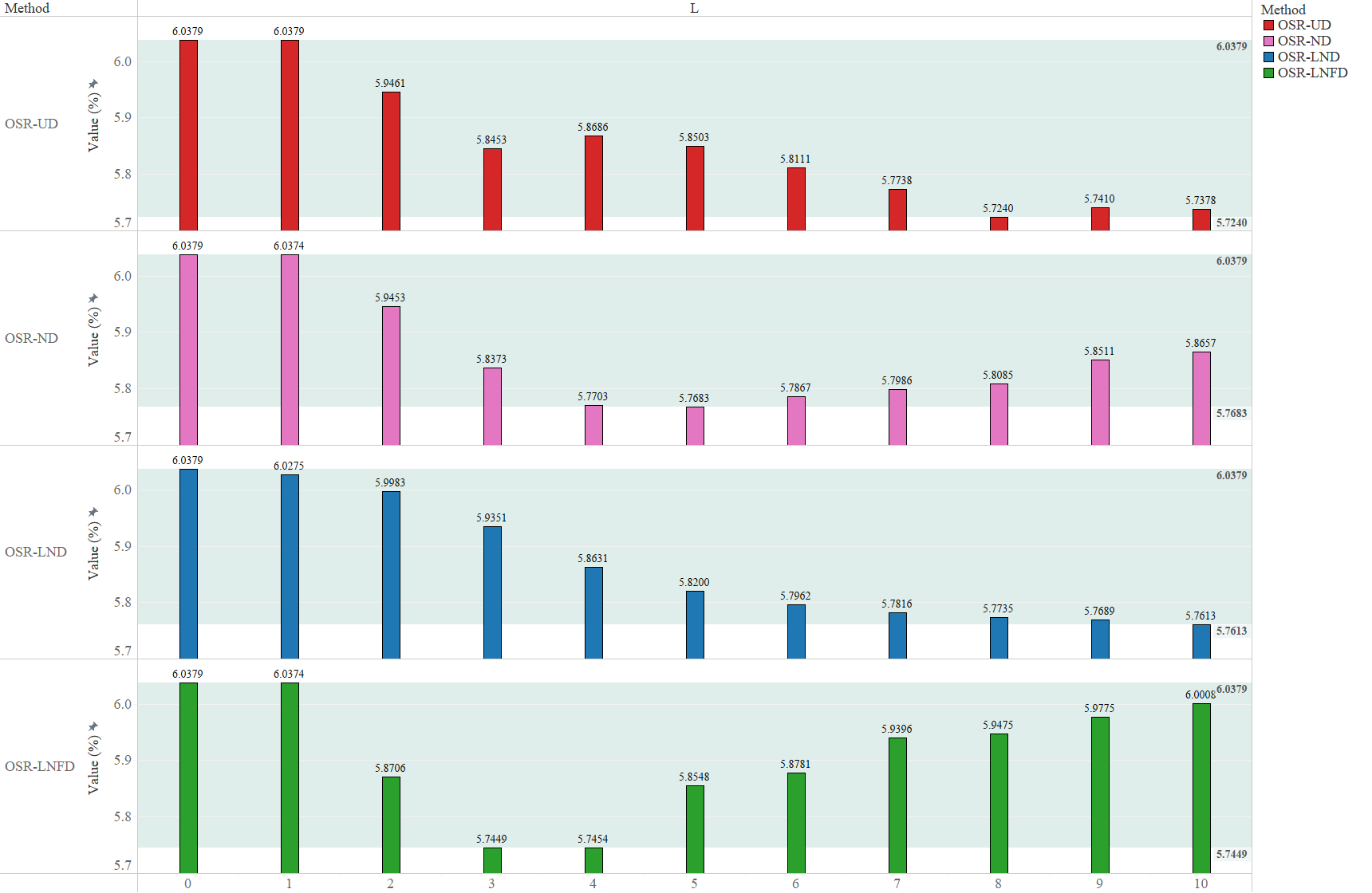

Supplement: Supplementary file 4 — Additional file 4. Fig. S3: MAPEs under different distribution patterns of intervention effect. [file 41256_2023_312_MOESM4_ESM.docx]

# Additional file


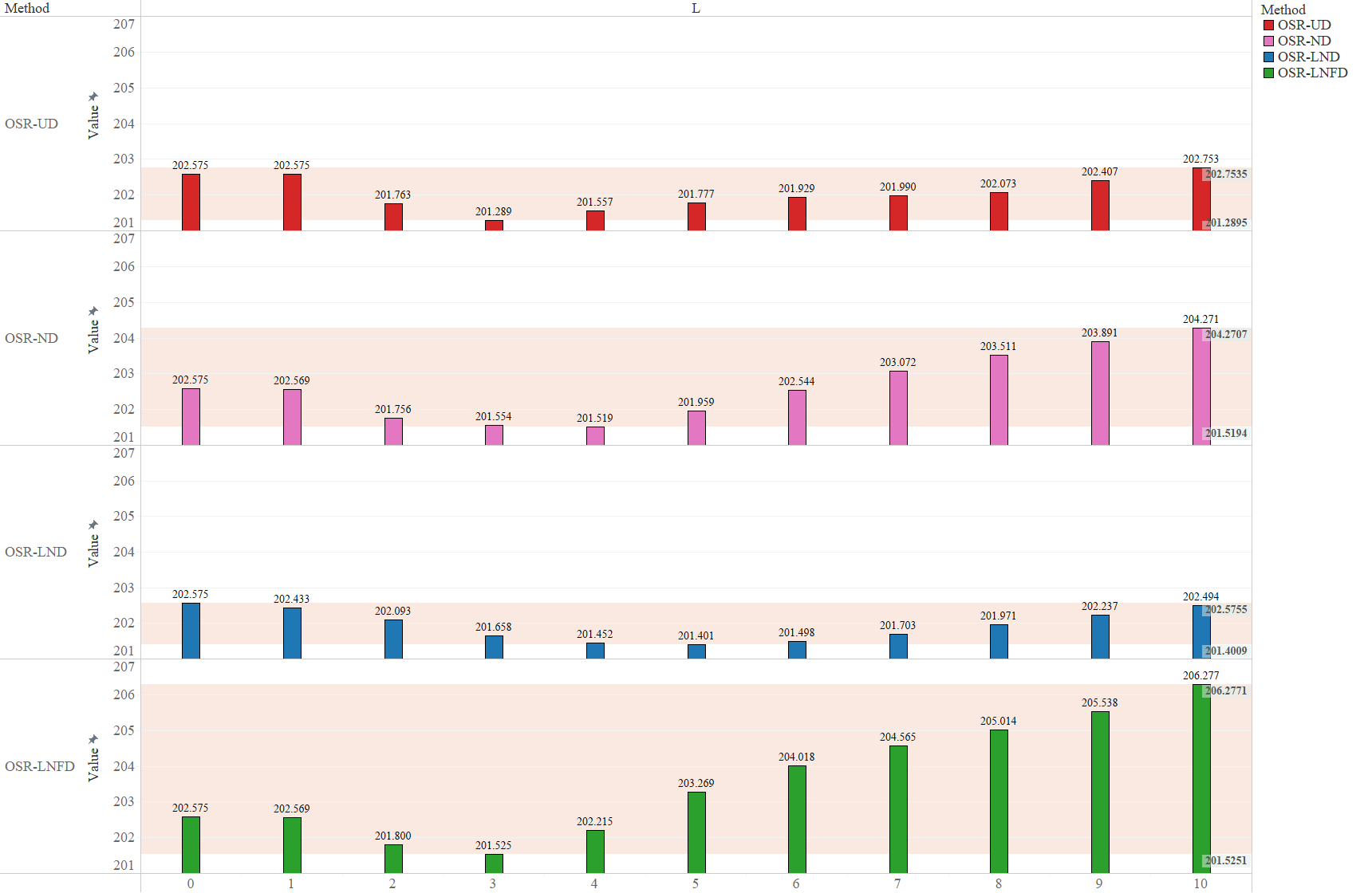

Supplement: Supplementary file 5 — Additional file 5. Fig. S4: MADs under different distribution patterns of intervention effect. [file 41256_2023_312_MOESM5_ESM.docx]
