# Supplementary material for: Optimized segmented regression models for the transition period of intervention effects
Source: Glob Health Res Policy. 2023 Jul 24;8:29. doi: 10.1186/s41256-023-00312-3 (PMC10364415; doi:10.1186/s41256-023-00312-3)
Supplement: Supplementary file 2 — Additional file 2. Fig. S1: Parameter estimation result planes and corresponding external studentized residuals. [file 41256_2023_312_MOESM2_ESM.docx]

| **Method** | **MSE** | **MAE** | **MAPE** | **MSD** |
| --- | --- | --- | --- | --- |
| **OSR-UD** | 8 | 8 | 8 | 3 |
| **OSR-ND** | 5 | 4 | 5 | 4 |
| **OSR-LND** | 10 | 10 | 10 | 5 |
| **OSR-LNDF** | 3 | 3 | 3 | 3 |
