# Supplementary material for: Optimized segmented regression models for the transition period of intervention effects
Source: Glob Health Res Policy. 2023 Jul 24;8:29. doi: 10.1186/s41256-023-00312-3 (PMC10364415; doi:10.1186/s41256-023-00312-3)
Supplement: Supplementary file 6 — Additional file 6. Table S2: ${\widehat{\beta }}_{0}$β^0 estimation results and corresponding 95% CIs. [file 41256_2023_312_MOESM6_ESM.docx]

| $\boldsymbol{L}$ | **OSR-UD** | **OSR-ND** | **OSR-LND** | **OSR-LNFD** |
| --- | --- | --- | --- | --- |
| 0 | 189.3106(178.5652,200.0561) | 189.3106(178.5652,200.0561) | 189.3106(178.5652,200.0561) | 189.3106(178.5652,200.0561) |
| 1 | 189.3106(178.5652,200.0561) | 189.3162(178.5728,200.0596) | 189.3162(178.5728,200.0596) | 189.3162(178.5728,200.0596) |
| 2 | 189.9828(179.6056,200.3599) | 189.9879(179.6135,200.3624) | 189.9517(179.7674,200.136) | 189.9517(179.7674,200.136) |
| 3 | 190.382(180.3212,200.4428) | 190.1604(180.0903,200.2306) | 190.1878(180.365,200.0105) | 190.1878(180.365,200.0105) |
| 4 | 190.1506(180.1358,200.1653) | 190.1909(180.341,200.0408) | 189.5979(179.7899,199.406) | 189.5979(179.7899,199.406) |
| 5 | 189.9632(180.0146,199.9117) | 189.8157(179.9989,199.6325) | 188.6897(178.7241,198.6552) | 188.6897(178.7241,198.6552) |
| 6 | 189.8379(179.9846,199.6912) | 189.3152(179.4627,199.1678) | 188.0418(178.0464,198.0373) | 188.0418(178.0464,198.0373) |
| 7 | 189.7967(180.0799,199.5136) | 188.8637(178.997,198.7304) | 187.5682(177.6327,197.5037) | 187.5682(177.6327,197.5037) |
| 8 | 189.7346(180.1447,199.3244) | 188.4895(178.6469,198.3321) | 187.1768(177.3502,197.0034) | 187.1768(177.3502,197.0034) |
| 9 | 189.4336(179.8475,199.0196) | 188.1636(178.371,197.9562) | 186.7058(176.9531,196.4584) | 186.7058(176.9531,196.4584) |
| 10 | 189.1178(179.5202,198.7153) | 187.8326(178.0892,197.576) | 186.0201(176.2252,195.8151) | 186.0201(176.2252,195.8151) |
