# Supplementary material for: Optimized segmented regression models for the transition period of intervention effects
Source: Glob Health Res Policy. 2023 Jul 24;8:29. doi: 10.1186/s41256-023-00312-3 (PMC10364415; doi:10.1186/s41256-023-00312-3)
Supplement: Supplementary file 7 — Additional file 7. Table S3: ${\widehat{\beta }}_{1}$β^1 estimation results and corresponding 95% CIs. [file 41256_2023_312_MOESM7_ESM.docx]

| $\boldsymbol{L}$ | **OSR-UD** | **OSR-ND** | **OSR-LND** | **OSR-LNFD** |
| --- | --- | --- | --- | --- |
| 0 | 0.2434(-0.0124,0.4992) | 0.2434(-0.0124,0.4992) | 0.2434(-0.0124,0.4992) | 0.2434(-0.0124,0.4992) |
| 1 | 0.2434(-0.0124,0.4992) | 0.2432(-0.0126,0.4989) | 0.2432(-0.0126,0.4989) | 0.2432(-0.0126,0.4989) |
| 2 | 0.2161(-0.0301,0.4624) | 0.2159(-0.0302,0.4621) | 0.2174(-0.0228,0.4576) | 0.2174(-0.0228,0.4576) |
| 3 | 0.2001(-0.0376,0.4379) | 0.2091(-0.0283,0.4464) | 0.208(-0.0221,0.4382) | 0.208(-0.0221,0.4382) |
| 4 | 0.2093(-0.0264,0.445) | 0.2079(-0.0231,0.4388) | 0.2315(0.0034,0.4596) | 0.2315(0.0034,0.4596) |
| 5 | 0.2168(-0.0164,0.45) | 0.2228(-0.0061,0.4517) | 0.2675(0.0375,0.4975) | 0.2675(0.0375,0.4975) |
| 6 | 0.2219(-0.0082,0.4519) | 0.2427(0.0142,0.4713) | 0.2931(0.0641,0.5221) | 0.2931(0.0641,0.5221) |
| 7 | 0.2237(-0.0023,0.4497) | 0.2607(0.033,0.4884) | 0.3119(0.0859,0.5378) | 0.3119(0.0859,0.5378) |
| 8 | 0.2264(0.0042,0.4486) | 0.2756(0.0497,0.5015) | 0.3273(0.1054,0.5492) | 0.3273(0.1054,0.5492) |
| 9 | 0.238(0.0167,0.4594) | 0.2886(0.0649,0.5122) | 0.3455(0.1269,0.5642) | 0.3455(0.1269,0.5642) |
| 10 | 0.2502(0.0293,0.471) | 0.3016(0.0802,0.523) | 0.2408(0.0154,0.4662) | 0.3717(0.1536,0.5898) |
