# Supplementary material for: Optimized segmented regression models for the transition period of intervention effects
Source: Glob Health Res Policy. 2023 Jul 24;8:29. doi: 10.1186/s41256-023-00312-3 (PMC10364415; doi:10.1186/s41256-023-00312-3)
Supplement: Supplementary file 8 — Additional file 8. Table S4: ${\widehat{\beta }}_{2}$β^2 estimation results and corresponding 95% CIs. [file 41256_2023_312_MOESM8_ESM.docx]

| $\boldsymbol{L}$ | **OSR-UD** | **OSR-ND** | **OSR-LND** | **OSR-LNFD** |
| --- | --- | --- | --- | --- |
| 0 | 52.854(34.2369,71.4712) | 52.854(34.2369,71.4712) | 52.854(34.2369,71.4712) | 52.854(34.2369,71.4712) |
| 1 | 52.854(34.2369,71.4712) | 52.9024(34.2834,71.5214) | 52.9024(34.2834,71.5214) | 52.9024(34.2834,71.5214) |
| 2 | 60.1312(41.4615,78.8008) | 60.1896(41.5182,78.861) | 62.4537(44.042,80.8655) | 62.4537(44.042,80.8655) |
| 3 | 66.3099(47.5686,85.0513) | 65.0153(46.5078,83.5229) | 69.0397(50.6087,87.4706) | 69.0397(50.6087,87.4706) |
| 4 | 69.1541(49.8359,88.4724) | 69.425(50.7867,88.0634) | 71.0807(52.0557,90.1057) | 71.0807(52.0557,90.1057) |
| 5 | 72.43(52.5447,92.3153) | 71.591(52.4684,90.7136) | 71.1764(51.1286,91.2242) | 71.1764(51.1286,91.2242) |
| 6 | 76.3092(55.8848,96.7337) | 73.0358(53.2559,92.8157) | 73.1096(52.2089,94.0103) | 73.1096(52.2089,94.0103) |
| 7 | 80.9808(60.0758,101.8857) | 74.8759(54.4409,95.3109) | 76.5754(54.9444,98.2064) | 76.5754(54.9444,98.2064) |
| 8 | 85.8777(64.4468,107.3086) | 77.397(56.3468,98.4472) | 81.1291(58.8181,103.4401) | 81.1291(58.8181,103.4401) |
| 9 | 89.686(67.417,111.955) | 80.5004(58.8535,102.1473) | 85.7099(62.5812,108.8386) | 85.7099(62.5812,108.8386) |
| 10 | 93.6112(70.4184,116.804) | 83.8829(61.6011,106.1646) | 78.788(58.2046,99.3713) | 89.1614(64.8612,113.4616) |
